# Reconstructing the Genetic Relationship between Ancient and Present-Day Siberian Populations

**DOI:** 10.1093/gbe/evae063

**Published:** 2024-03-25

**Authors:** Haechan Gill, Juhyeon Lee, Choongwon Jeong

**Affiliations:** School of Biological Sciences, Seoul National University, Seoul 08826, Republic of Korea; School of Biological Sciences, Seoul National University, Seoul 08826, Republic of Korea; School of Biological Sciences, Seoul National University, Seoul 08826, Republic of Korea

**Keywords:** Siberian ancestry, Syalakh–Belkachi cultures, Yakutia, Baikal, ancient genome

## Abstract

Human populations across a vast area in northern Eurasia, from Fennoscandia to Chukotka, share a distinct genetic component often referred to as the Siberian ancestry. Most enriched in present-day Samoyedic-speaking populations such as Nganasans, its origins and history still remain elusive despite the growing list of ancient and present-day genomes from Siberia. Here, we reanalyze published ancient and present-day Siberian genomes focusing on the Baikal and Yakutia, resolving key questions regarding their genetic history. First, we show a long-term presence of a unique genetic profile in southern Siberia, up to 6,000 yr ago, which distinctly shares a deep ancestral connection with Native Americans. Second, we provide plausible historical models tracing genetic changes in West Baikal and Yakutia in fine resolution. Third, the Middle Neolithic individual from Yakutia, belonging to the Belkachi culture, serves as the best source so far available for the spread of the Siberian ancestry into Fennoscandia and Greenland. These findings shed light on the genetic legacy of the Siberian ancestry and provide insights into the complex interplay between different populations in northern Eurasia throughout history.

**Significance**

The investigation into the origin and spread of Siberian ancestry, a unique genetic component found in human populations spanning from Fennoscandia to Chukotka, has been understudied. To address this gap, we delve into the population dynamics of Middle Holocene Siberian hunter–gatherers and establish the connections between present-day and Middle Holocene Siberian populations. Significantly, we find that the Middle Neolithic Yakutian Belkachi culture played a pivotal role in the spread of the Siberian ancestry.

## Introduction

Migration and admixture are key demographic events that have influenced the genetic structure of modern human populations (Patterson et al. 2012). The genetic diversity of inhabitants in Inner Eurasia, a vast geographic region encompassing Siberia and the Eurasian Steppe, has been shaped by a complex history of mixture between diverse source populations of both eastern and western Eurasian origins (Jeong et al. 2019). As a result of this complex history, present-day Inner Eurasian populations are stratified into three distinct admixture clines mirroring geography. The northernmost one among these clines, composed of populations from the boreal forest and tundra regions who mostly speak the Uralic and Yeniseian languages, share a distinct type of Eastern Eurasian ancestry, frequently referred to as the Siberian ancestry in recent archeogenetics literature (Tambets et al. 2018). Among the present-day populations, it is most enriched in Nganasans and other Samoyedic-speaking ones such as Nenets, Enets, and Selkups.

The Samoyedic-speaking populations inhabit the northernmost region of Siberia (Nganasans, Enets, and Nenets) as well as the Yenisei River basin to the south (Selkups). Although ancient genomes have been only scarcely reported in these regions, the Siberian ancestry was present in a larger area in the past, including early Metal Age individuals from Bolshoy Oleni Ostrov in the Kola Peninsula, Iron Age individuals from the eastern Baltic Sea, and Iron Age individuals from the Volga–Oka interfluve (Lamnidis et al. 2018; Saag et al. 2019; Peltola et al. 2023). Together with the genetic analysis of present-day populations, these studies suggest that the populations with the Siberian ancestry once occupied a large area in Siberia and northeastern Europe and formed a substratum for the genetic profile of present-day populations in the regions. Therefore, it is crucial to trace the origins and spreads of the Siberian ancestry for understanding the formation of present-day human populations and languages in northern Eurasia. Despite the recent accumulation of ancient genome data in Siberia, centered on southern Siberia, it remains obscure how these populations are related to present-day inhabitants of Siberia, such as Nganasans, calling for a careful reinvestigation of previously published data.

Among the previously published ancient genomes, the Middle Holocene (ca. 8,000 to 3,000 yr ago; Sandweiss et al. 1999) southern Siberians represent a pivotal ancient lineage connected to the present-day Siberian ancestry. Of particular interest are those from the archeological sites at Lake Baikal and Yakutia because they harbored Y haplogroups N and Q, which are prevalent in present-day Siberians (Karafet et al. 2018). These archeological sites also belong to multiple interrelated but distinct archeological cultures of hunter–gatherers: the Lake Baikal region was inhabited by the Kitoi culture during the Early Neolithic period (ca. 8,000 to 6,800 yr ago), followed by the Serovo–Glazkovo culture during the Late Neolithic period and Early Bronze Age (ca. 6,000 to 3,400 yr ago), while the Yakutia region was inhabited by the Syalakh–Belkachi culture during the Early–Middle Neolithic periods (ca. 7,000 to 4,300 yr ago), succeeded by the Late Neolithic Ymyyakhtakh culture (ca. 4,300 to 3,300 yr ago; Weber and Bettinger 2010; Coutouly 2016). Overall, they show genome-wide genetic profiles closely related to present-day Siberians but still different enough to reject a simple ancestor–descendant relationship. Therefore, the Middle Holocene Siberians in Yakutia and Lake Baikal provide an excellent starting point from which we can build a historical model for the origins of the Siberian ancestry and populations harboring it.

**Fig. 1.**
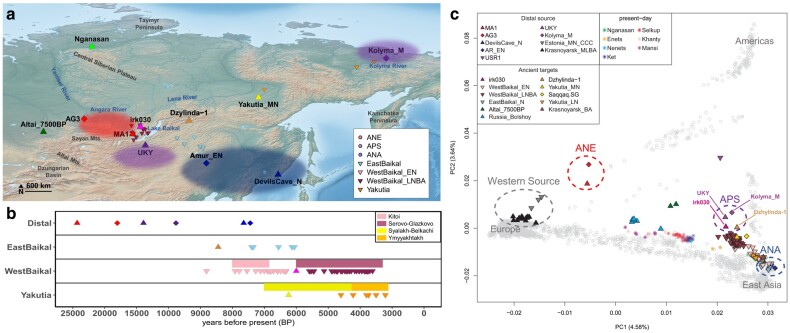
a) Geographic sites of analyzed samples in this study. Three distal source populations (AG3, MA1 for ANE; UKY, Kolyma_M for APS; and Amur_EN, DevilsCave_N for ANA), which are used for explaining the formation of Middle Holocene Siberians, are highlighted by color-shaded ellipses on the map. Among Middle Holocene Siberians, key individuals, irk030, Dzhylinda-1, Altai_7500BP, and Yakutia_MN, are marked separately, and the rest of the Middle Holocene and present-day populations are marked based on geographic locations and archeological period. The base map data are downloaded from Natural Earth (https://www.naturalearthdata.com/downloads/). b) The radiocarbon dating of three distal sources and Middle Holocene Siberians. The dating results of key ancient samples are presented in before the present (BP). c) PCA performed with present-day Eurasian and American individuals. Each present-day individual is placed on PC 1 and 2 coordinates by colored asterisk symbols (key Siberian populations) or gray circles (others). Key ancient individuals are projected on precalculated PCs and marked with color-filled polygon symbols.

The genetic makeup of the Middle Holocene Siberians resulted from the admixture of three ancestries (Fig. 1): Ancient North Eurasian (ANE), Ancient Paleo-Siberian (APS; an ancestry closely related to the Native American ancestries), and Ancient North Asian (ANA). ANE ancestry is represented by the Upper Paleolithic individuals from the Mal’ta (MA1) and Afontova Gora sites (AG2 and AG3; Raghavan et al. 2014; Fu et al. 2016). During the Last Glacial Maximum (LGM), ANE ancestry intermixed with populations of Eastern Eurasian origin and formed the ancestral population of Native Americans. This ancestral population left its genetic legacy in later APS populations, e.g. 14,000-yr-old terminal Pleistocene individual from Ust-Kyakta-3 in southern Siberia (UKY) and 9,800-yr-old Mesolithic individual from the Duvanny Yar site at the Kolyma River in northern Siberia (Kolyma_M; Sikora et al. 2019; Yu et al. 2020). At the beginning of the Middle Holocene, individuals of ANA ancestry already appeared in both East and West Baikal (Kılınç et al. 2021), presumably expanded from the neighboring regions in northeastern China where ANA ancestry was present at least 14,000 yr ago (Siska et al. 2017; Ning et al. 2020; Mao et al. 2021). Although the genetic profile and the geographic distribution of these three ancestries in Siberia have been actively investigated using ancient genomes, it remains unexplored how, when, and where the genetic profiles of present-day Siberians were formed out of these three ancestral populations. In this study, we provide a proximal historical model for the genetic relationship between a comprehensive set of published Siberian genome data. Our findings demonstrate that the APS population was present in the Baikal and Yakutia during the Middle Holocene, and in each region, the local APS population formed a genetic substratum for the later populations. Yakutian populations from three different time points are genetically distinct due to multiple streams of ANA-related gene flow between them. Finally, we show that the Middle Neolithic Yakutia (Yakutia_MN) population serves as a better-fitting source than the Lake Baikal one for the Siberian ancestry found in the genetic makeup of northern Siberians, northeastern Europeans, Paleo-Eskimos, and ancient Athabaskans.

## Results

### The Genetic Profile of Ancient Siberian Individuals

We curated published genomes and genome-wide data of ancient Siberian populations focusing on Lake Baikal and Yakutia. Most ancient individuals date to the Middle Holocene, ranging 8,800 to 3,100 BP (Fig. 1). To overview the genetic profile of these ancient individuals, we performed principal component analysis (PCA; Patterson et al. 2006) and projected them onto the principal components (PCs) calculated from 2,270 present-day Eurasian and American individuals (Fig. 1). PC1 separates individuals from west to east, and PC2 separates from Eurasians to Native Americans. While most Middle Holocene Siberian individuals fall on a cline in the PC space between the ANE and ANA populations, two individuals deviate from this cline: Dzhylinda-1, the earliest individual among east Baikal individuals (6,564 to 6,429 cal. BCE), and irk030, the earliest Late Neolithic West Baikal individual (4,150 to 3,950 cal. BCE; Kılınç et al. 2021). They are shifted upward along PC2, suggesting an extra affinity with Native Americans.

For the group-based analyses, we removed PCA outliers and first-degree relatives and allocated the remaining ancient Siberian individuals into five analysis supergroups according to their geographic location, archeological period, and PCA pattern: Early Neolithic East Baikal (EastBaikal_N; *n* = 5), Early Neolithic West Baikal (WestBaikal_EN; *n* = 21), Late Neolithic to Early Bronze Age West Baikal (WestBaikal_LNBA; *n* = 45), Middle Neolithic Yakutia (Yakutia_MN; *n* = 1), and Late Neolithic Yakutia (Yakutia_LN; *n* = 5; de Barros Damgaard, Martiniano, et al. 2018; Sikora et al. 2019; Yu et al. 2020; Kılınç et al. 2021; supplementary table S1, Supplementary Material online).

### The Ancestral Native American Genetic Legacy Diverged in Holocene Siberia

**Fig. 2.**
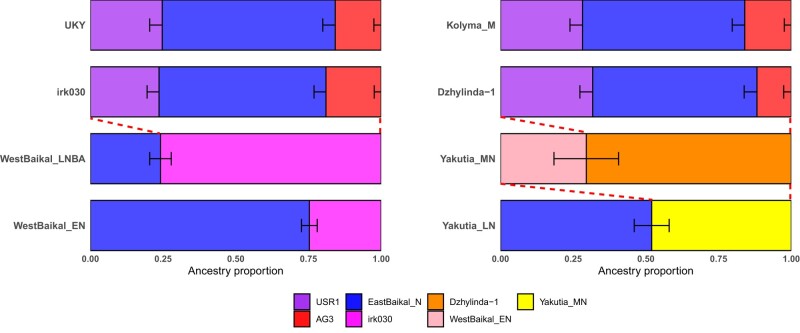
The genetic profiles of ancient Siberians estimated by *qpAdm*. Target populations are modeled as a mixture of two or three populations. The ancestry proportion of each source is represented by the box size on the *x* axis. Horizontal bars represent ±1 SE estimated by *qpAdm* using 5 cM block jackknifing. Detailed results are presented in supplementary tables S4 and S5, Supplementary Material online.

We first model the genetic profile of the two ancient Siberian individuals, Dzhylinda-1 and irk030, who potentially show a link to the ancestral Native American gene pool (Fig. 1). Of note, two earlier-period APS individuals, 14,000-yr-old UKY and 9,800-yr-old Kolyma_M, show a similar shift in the PC space toward Native Americans to a greater degree. We formally tested if an ancestry component related to Native Americans is required to explain the genetic profile of these Siberian individuals using *qpAdm* (Lazaridis et al. 2016). While the two-way admixture models of neither ANE + ANA nor Native American + ANA fit them, the three-way model of ANE + ANA + Native American adequately fits them with similar ancestry proportions with the earlier APS individuals (Fig. 2; supplementary table S4, Supplementary Material online). Indeed, using *qpWave* (Reich et al. 2012), we show that (UKY, Kolyma_M, irk030) and (UKY, Kolyma_M, Dzhylinda-1) can be modeled as a clade, respectively (supplementary table S4, Supplementary Material online). However, Dzhylinda-1 and irk030 do not form a clade in *qpWave* analysis, suggesting a difference in the admixture proportions between these two Holocene individuals. Our analysis extends the presence of the APS population at least to the Late Neolithic (irk030) in Siberia.

By utilizing outgroup-*f*_3_ statistics (Patterson et al. 2012) in the form of *f*_3_(Mbuti; irk030/Dzhylinda-1, X), we searched for the genetic link of irk030 and Dzhylinda-1 with later populations (supplementary fig. S2 and table S8, Supplementary Material online). Dzhylinda-1 and irk030 showed a high genetic affinity with Yakutia_MN and WestBaikal_LNBA, respectively. *f*_4_ statistics (Patterson et al. 2012) in the form of *f*_4_(Mbuti, irk030/Dzhylinda-1; WestBaikal_LNBA, Yakutia_MN) confirm that Dzhylinda-1 and irk030 are closer to Yakutia_MN and WestBaikal_LNBA, respectively (supplementary fig. S3 and table S7, Supplementary Material online). Notably, irk030 and Dzhylinda-1 are the only populations that precede WestBaikal_LNBA and Yakutia_MN in their regions, respectively, and distinguish them. These findings suggest that the difference between the Yakutia and West Baikal populations trace back to the distinct Middle Holocene APS populations represented by Dzhylinda-1 and irk030, respectively. We note that Dzhylinda-1 is from the northern part of the East Baikal region, close to but not in Yakutia, but utilize him for modeling Yakutian populations based on this genetic affinity.

### The Late Neolithic Local APS Population in West Baikal

The genetic profile of the West Baikal populations underwent a transition from ANA-rich WestBaikal_EN population to APS-rich WestBaikal_LNBA, coinciding with the cultural shift from the Kitoi to the Serovo–Glazkovo culture (Weber et al. 2002). Previous studies have suggested that the resurgence of the APS ancestry in Serovo–Glazkovo was due to gene flow from an APS source like UKY, Kolyma_M, and Altai hunter–gatherers (Altai_HG; Sikora et al. 2019; Yu et al. 2020; Wang et al. 2023). Utilizing irk030, the earliest Serovo–Glazkovo-related individual with the APS genetic profile distinct from later Serovo–Glazkovo ones, we examined the so far unexplored hypothesis that irk030 represents the source of the APS ancestry found in the West Baikal populations.

Using *f*_4_ symmetry test, we found that irk030 has higher affinity with the ANE ancestry represented by AG3 and Tarim_EMBA1, while other West Baikal populations, both the Early Neolithic Kitoi and the later Serovo–Glazkovo ones, have more ANA ancestry represented by East Baikal populations (supplementary fig. S3, Supplementary Material online; Table 1; supplementary table S7, Supplementary Material online). We further explored the genetic makeup of the West Baikal populations by investigating the admixture model between ANA and irk030 and found EastBaikal_N + irk030 adequately fit both West Baikal populations (Fig. 2; supplementary table S5, Supplementary Material online). WestBaikal_EN showed a relatively lower contribution from irk030 (*P* = 0.088; 25% from irk030), while WestBaikal_LNBA showed a higher contribution from irk030 (*P* = 0.289; 76% from irk030; Fig. 2 and Table 1; supplementary table S5, Supplementary Material online). Importantly, previously reported admixture models for the West Baikal populations, using Altai_HG as the APS source instead of irk030, turn infeasible when irk030 is added to the outgroup population set following the *qpAdm* rotating approach (Harney et al. 2021; *P* < 0.004; supplementary table S5, Supplementary Material online). Additionally, we tested if irk030 provides a better APS proxy for the APS ancestry in Altai_HG, who were modeled as a mixture of ANE and APS (Wang et al. 2023). We show that Altai_HG is adequately modeled as irk030 + ANE while APS + ANE models with other APS sources break when irk030 is added to the outgroup population set (supplementary table S5, Supplementary Material online). Based on these results, we propose that the connection between West Baikal and the Altai was mediated by a gene flow of the APS ancestry from West Baikal to the Altai, flipping the direction of gene flow previously suggested (Wang et al. 2023).

**Table 1:** Key genetic symmetricity test and admixture modeling results

Key *f*_4_ statistic results between target populations						
pop1	pop2	pop3	pop4	*f*_4_	*Z*-score	Table
Mbuti	irk030	WestBaikal_LNBA	Yakutia_MN	−0.00187	−3.833	S7
Mbuti	Dzhylidna-1	WestBaikal_LNBA	Yakutia_MN	0.00190	3.256	S7
Mbuti	EastBaikal_N	irk030	WestBaikal_EN	0.00487	13.032	S7
Mbuti	EastBaikal_N	irk030	WestBaikal_LNBA	0.00239	6.557	S7
Mbuti	WestBaikal_EN	Dzhylinda-1	Yakutia_MN	0.00161	3.356	S7
Mbuti	EastBaikal_N	Yakutia_MN	Yakutia_LN	0.00238	5.525	S7

Key *qpAdm* and *qpWave* results of target populations								
Target	ref1	ref2	ref3	coeff1	coeff2	coeff3	*P*	Table
irk030	UKY	…	…	…	…	…	0.085	S4
WestBaikal_EN	irk030	EastBaikal_N	…	0.247 ± 0.027	0.753 ± 0.027	…	0.088	S5
WestBaikal_LNBA	irk030	EastBaikal_N	…	0.759 ± 0.037	0.241 ± 0.037	…	0.289	S5
Dzhylinda-1	UKY	…	…	…	…	…	0.911	S4
Yakutia_MN	Dzhylinda-1	WestBaikal_EN	…	0.705 ± 0.111	0.295 ± 0.111	…	0.235	S5
Yakutia_LN	Yakutia_MN	EastBaikal_N	…	0.480 ± 0.060	0.520 ± 0.060	…	0.942	S5
Krasnoyarsk_BA	Yakutia_MN	EastBaikal_N	…	0.543 ± 0.092	0.457 ± 0.092	…	0.675	S6
Nganasan	Yakutia_MN	EastBaikal_N	…	0.590 ± 0.057	0.410 ± 0.057	…	0.920	S6
Enets	Yakutia_MN	Krasnoyarsk_MLBA	…	0.884 ± 0.019	0.116 ± 0.019	…	0.681	S6
Nenets	Yakutia_MN	Krasnoyarsk_MLBA	…	0.748 ± 0.021	0.252 ± 0.021	…	0.084	S6
Selkup	Yakutia_MN	Krasnoyarsk_MLBA	irk030	0.364 ± 0.074	0.268 ± 0.010	0.367 ± 0.071	0.886	S6
Ket	Yakutia_MN	Krasnoyarsk_MLBA	irk030	0.346 ± 0.094	0.218 ± 0.011	0.436 ± 0.090	0.290	S6
Mansi	Yakutia_MN	Krasnoyarsk_MLBA	…	0.577 ± 0.012	0.423 ± 0.012	…	0.714	S6
Khanty	Yakutia_MN	Krasnoyarsk_MLBA	…	0.600 ± 0.013	0.400 ± 0.013	…	0.146	S6
Russia_Bolshoy	Yakutia_MN	Estonia_MN_CCC	…	0.506 ± 0.018	0.494 ± 0.018	…	0.309	S6
Saqqaq	Yakutia_MN	…	…	…	…	…	0.150	S6
Athabaskan_1100BP	Yakutia_MN	Anzick	…	0.379 ± 0.028	0.621 ± 0.028	…	0.233	S6
	Saqqaq	Anzick	…	0.370 ± 0.028	0.630 ± 0.028	…	0.517	S6
Ekven_IA	Yakutia_MN	Anzick	…	0.606 ± 0.021	0.394 ± 0.021	…	0.308	S6
	Saqqaq	Anzick	…	0.607 ± 0.020	0.393 ± 0.020	…	0.932	S6

*f*_4_ (pop1, pop2, pop3, and pop4) for key genetic tests. *Z*-scores were calculated by dividing *f*_4_ by the s.e.m. estimated by 5 cM block jackknifing. Key qpAdm and qpWave results: coeff1, coeff2, and coeff3 represent the ancestry proportion (±1 s.e.m.) contributed by each reference population. The “*P*” column indicates the *P* value, and the “Table” column indicates the supplementary table that includes the result.

### Repeated Introduction of ANA Ancestry into Yakutia Populations

In Yakutia, we first show that Dzhylinda-1, Yakutia_MN, and Yakutia_LN do not form a simple time series of local population continuity without gene flow from other sources. While a close relationship between Dzhylinda-1 and Yakutia_MN was suggested in the previous study, their relationship was not formally modeled (Kılınç et al. 2021). We show that WestBaikal_EN is closer to Yakutia_MN than its preceding Dzhylinda-1 as shown in *f*_4_(Mbuti, WestBaikal_EN; Dzylinda-1, Yakutia_MN) = 3.4 standard error measures (s.e.m.; supplementary fig. S3 and table S7, Supplementary Material online). Likewise, many ANA-related populations are closer to Yakutia_LN than its preceding Yakutia_MN, e.g. *f*_4_(Mbuti, EastBaikal_N; Yakutia_MN, Yakutia_LN) = 5.5 s.e.m. (supplementary fig. S3 and table S7, Supplementary Material online). However, ancient West Baikal populations are symmetrically related to Yakutia_MN and Yakutia_LN: *f*_4_(Mbuti, WestBaikal_EN/WestBaikal_LNBA; Yakutia_MN, Yakutia_LN) = 2.6 and 1.4 s.e.m., respectively (supplementary table S7, Supplementary Material online). Formally modeling this relationship with *qpAdm*, we show that Yakutia_MN and Yakutia_LN are adequately modeled as Dzhylinda-1 + WestBaikal_EN (*P* = 0.235; 30% contribution from WestBaikal_EN) and Yakutia_MN + EastBaikal_N (*P* = 0.942; 52% contribution from EastBaikal_N), respectively (Fig. 2 and Table 1; supplementary table S5, Supplementary Material online). In summary, our findings illustrate three distinct genetic strata in Yakutia, represented by a time series from Dzhylinda-1 to Yakutia_MN to Yakutia_LN, involving multiple episodes of ANA ancestry introduction.

**Fig. 3.**
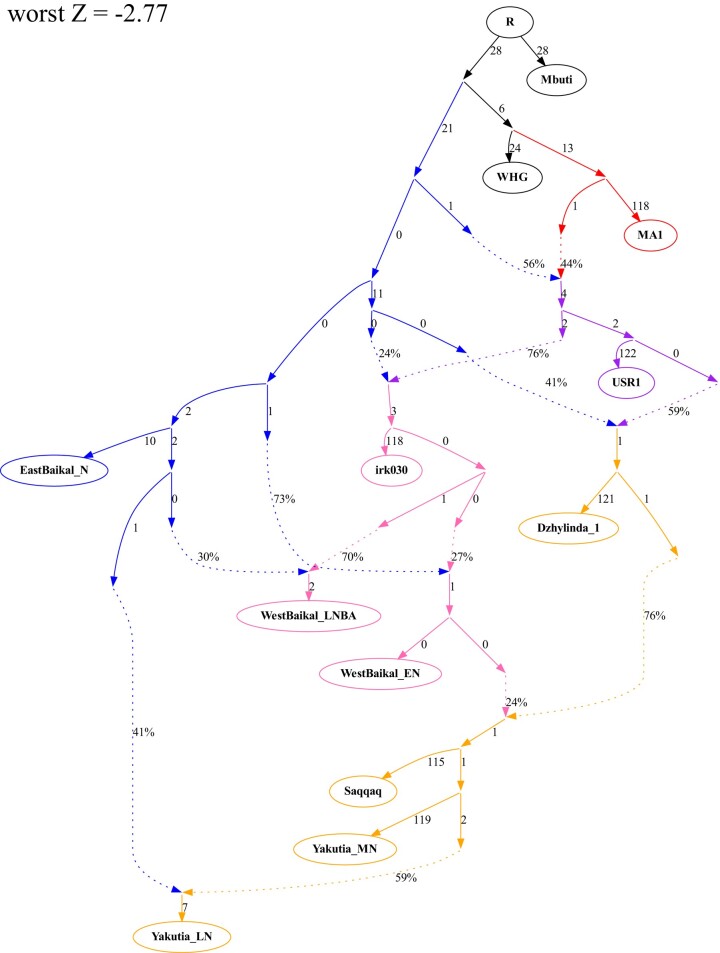
Manually fitted admixture graph explaining population dynamics of ancient Siberian populations. The admixture graph is based on proximal admixture modeling results and manually fitted by *qpGraph* function. ANE, ANA, Native American, West Baikal, and Yakutia lineages are shown in red, blue, purple, light pink, and yellow, respectively. The worst *Z*-score between fitted and observed *f*_4_ statistics is −2.77.

Lastly, we comprehensively tested all proximal models of the Middle Holocene Siberian populations by graph-based analysis, *qpGraph* (Fig. 3). We construct a basal graph including Mbuti, MA1, Western European hunter–gatherers (WHG), EastBaikal_N, and USR1, referring to the previous study (Yu et al. 2020). We added irk030 and Dzhylinda-1 as an independent mixture of Native American and ANA branches. Then, we modeled West Baikal and Yakutia populations as successors of irk030 and Dzhylinda-1, respectively. The proximal admixture models are well explained in the statistically feasible final admixture graph (worst *Z*-score = −2.77). We caution that we did not perform a comprehensive search over possible graph topologies and that the presented graph includes several zero-length branches. We speculate that this is likely due to lack of statistical resolution due to limited number of ancient genomes per group but may also reflect the demographic history of rapid branching events, such as a rapid expansion of the ANA-related population into Siberia.

### The Siberian Ancestry Originated from Neolithic Yakutia Populations

**Fig. 4.**
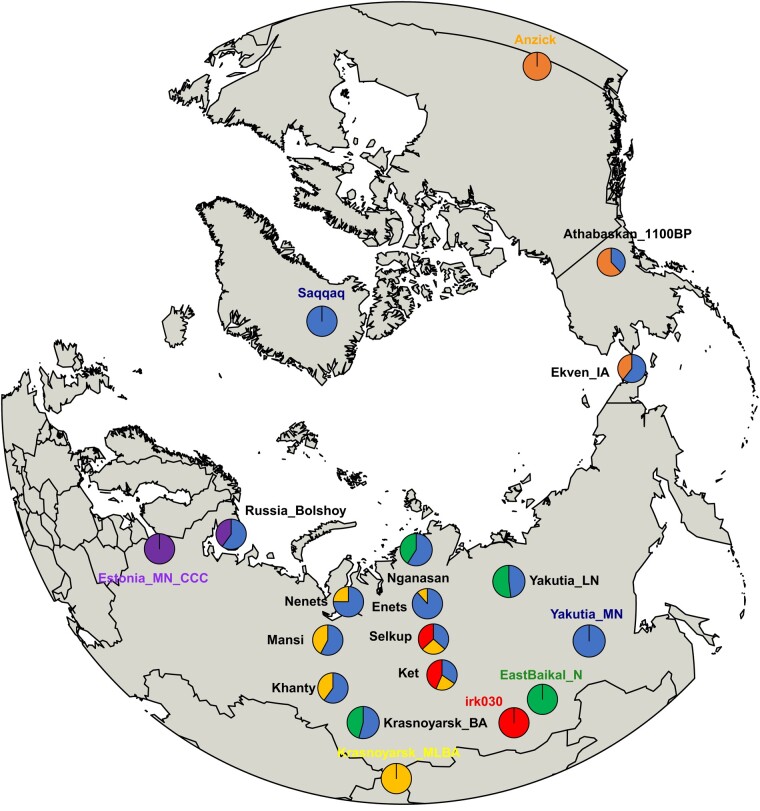
The genetic profiles of Siberian ancestry–related populations on a worldwide map. Yakutia_MN is used to represent Siberian ancestry, and each target is modeled as a mixture of Yakutia_MN and its local proximal source. All admixture models are feasible, and ancestry proportion is shown in pie charts (supplementary table S6, Supplementary Material online).

Our detailed examination of the Middle Holocene Siberian populations has provided significant insights into the origin of the Siberian ancestry. We identified two primary lineages within the Middle Holocene Siberians: the Lake Baikal lineage and the Yakutia lineage. Interestingly, we observed an increasing affinity between ancient Yakutia populations and present-day Nganasan over time, while this trend was not observed in the West Baikal populations (supplementary fig. S1, Supplementary Material online). Further analysis using the outgroup-*f*_3_ statistic revealed a strong genetic affinity between Nganasan and Yakutia, as well as a closely related individual in southern Siberia, Krasnoyarsk_BA (supplementary fig. S4 and table S9, Supplementary Material online). Similar to Yakutia_LN and Krasnoyarsk_BA, Nganasan could be modeled as Yakutia_MN + EastBaikal_N (*P* = 0.675; 54% contribution from Yakutia_MN), but its Yakutia_MN ancestry proportion was slightly higher than that of Yakutia_LN (48%; Fig. 4 and Table 1; supplementary table S6, Supplementary Material online). This model breaks when Yakutia_LN was added to the outgroup population set, supporting a strong affinity between Nganasan and Yakutia_LN (*P* = 2.38 × 10^−11^; supplementary table S6, Supplementary Material online). Therefore, we suggest that Nganasan descended from a metapopulation to which Yakutia_LN and Krasnoyarsk_BA belonged but its direct ancestor had less contribution from the EastBaikal_N-related gene flow than Yakutia_LN.

To understand better the genetic legacy of ancient Yakutian populations in present-day Siberians, we explored the admixture models of various present-day Siberian populations, including Enets, Nenets, Selkups, Kets, Mansi, and Khanty. Previous studies modeled these populations as either two-way admixture of Nganasan + Srubnaya (Jeong et al. 2019) or three-way admixture of Kolyma_M + DevilsCave_N + Afanasievo (Sikora et al. 2019). However, the former used present-day Nganasan as a source and the latter provided models with three or more sources only. In contrast, we find that a simple two-way admixture model of Yakutia_MN + Krasnoyarsk_MLBA fits all of the above six populations (*P* > 0.08; Fig. 4; supplementary table S6, Supplementary Material online). Competing models with earlier Yakutian source (Dzhylinda-1 + Krasnoyarsk_MLBA; *P* < 0.034) or with a later one (Yakutia_LN + Krasnoyarsk_MLBA; *P* < 0.006) uniformly fail (supplementary table S6, Supplementary Material online).

For Selkups and Kets who live most closely to the Baikal region among the above, the Yakutia_MN + Krasnoyarsk_MLBA model turned infeasible when WestBaikal_LNBA was included in outgroups (*P* < 0.006; supplementary table S6, Supplementary Material online). To quantify the WestBaikal_LNBA-related ancestry component in Selkups and Kets, we applied a three-way admixture model of Yakutia_MN + irk030 + Krasnoyarsk_MLBA, considering that WestBaikal_LNBA and Yakutia_MN are too similar to be used together as sources while keeping the resolution of the model sufficiently high. The indirect model with irk030 fits both Selkups and Kets adequately even when WestBaikal_LNBA is included as an extra outgroup with 37% to 44% contribution from irk030 (*P* > 0.289; Fig. 4; supplementary table S6, Supplementary Material online). The irk030-related contribution was not detected in the other four populations (supplementary table S6, Supplementary Material online).

While Yakutia_LN does not provide fitting models for the above-listed present-day Siberian populations, all present-day Siberian populations have higher outgroup-*f*_3_ values with Yakutia_LN/Nganasan than with the sources in the model, Yakutia_MN and Krasnoyarsk_MLBA (supplementary fig. S4 and tables S8 and S9, Supplementary Material online). Based on our results, we hypothesize that the true source of the Siberian ancestry in these populations likely stemmed from an unsampled population more closely related to Yakutia_LN/Nganasan than the sampled Yakutia_MN is, but not sharing the ANA gene flow with Yakutia_LN/Nganasan.

### Dispersal of the Middle Neolithic Yakutian Ancestry beyond Siberia

The Siberian ancestry extends beyond the Ural Mountains into northeastern European populations, indicating a historical migration from Siberia to the west. To pinpoint the genetic strata from which Siberian ancestry expanded and initially appeared in northeastern Europe, we examined early Metal Age individuals in Bolshoy Oleni Ostrov (Russia_Bolshoy). Russia_Bolshoy displayed high outgroup*-f*_3_ values with Tarim_EMBA1, Yakutia populations, and Eastern European hunter–gatherers (EHG; supplementary fig. S4 and table S8, Supplementary Material online). A parsimonious two-way admixture model, Yakutia_MN + Middle Neolithic Combed Ceramic Culture (Estonia_MN_CCC), adequately explains Russia_Bolshoy (*P* = 0.309; 51% contribution from Yakutia_MN; Fig. 4 and Table 1; supplementary table S6, Supplementary Material online). It is worth noting that Russia_Bolshoy exhibits a different major Y haplogroup pattern from Yakutia_MN (i.e. two Russia_Bolshoy males have Y haplogroup N, while Yakutia_MN has Y haplogroup Q; supplementary table S1, Supplementary Material online). We keep a possibility open that this is simply due to limited sampling of Yakutia_MN and suggest that further sampling of ancient genomes from Yakutia_MN is essential to fully understand the origins of Y haplogroup N in Russia_Bolshoy.

Regarding the origins of Paleo-Eskimos in Greenland, it has been suggested that they shared a cultural similarity with the Belkachi culture to which Yakutia_MN belongs (Powers and Jordan 1990; Coutouly 2016; Flegontov et al. 2019; Kılınç et al. 2021). However, the genetic connection between Belkachi and Paleo-Eskimo has not been thoroughly explored, especially in comparison to the earlier APS populations (Kılınç et al. 2021). We confirm that Yakutia_MN, who belonged to the Belkachi culture, is cladal with a Paleo-Eskimo individual Saqqaq even when earlier APS populations are included in the outgroups (*P* > 0.086; Table 1; supplementary table S6, Supplementary Material online). In addition, we confirm that Yakutia_MN can replace Saqqaq as a source in previously reported models for Athabaskans and Neo-Eskimos with comparable ancestry proportions: Yakutia_MN + Anzick fits ancient Athabaskans (Flegontov et al. 2019; *P* = 0.233; 38% contribution from Yakutia_MN) and Neo-Eskimos from Ekven site (Sikora et al. 2019; Wang et al. 2023; *P* = 0.308; 61% contribution from Yakutia_MN; Fig. 4; supplementary table S6, Supplementary Material online).

## Discussion

In this study, we reconstruct detailed population dynamics of ancient Siberian populations and propose the Neolithic Yakutia populations as the origin of the Siberian ancestry. While ancient individuals with the APS ancestry are sporadically found across Siberia from Baikal to Arctic Siberia, it remains unclear how the APS populations related to each other and to later populations in the region (Sikora et al. 2019; Yu et al. 2020). By showing that two ancient individuals, irk030 and Dzhylinda-1, belong to the APS metapopulation, we confirm a long-term presence of the APS population in southern Siberia between 14,000 and 6,000 yr ago. Moreover, these two individuals represent two sublineages within the APS metapopulation, forming the genetic substratum for the later Siberian populations in the West Baikal and Yakutia, respectively, thus suggesting that the divergence between the two regions dates back at least ca. 8,500 yr ago. However, it is worth noting that this divergence does not mean a complete separation between the two regions, as shown by a gene flow from WestBaikal_EN to Yakutia_MN, indicating an expansion of Kitoi culture–related populations to the Yakutia region. Such a genetic connection aligns with archeological studies (McKenzie 2009; Kuzmin 2015) describing the spread of net-impressed pottery style from West Baikal to Yakutia.

Contrary to previous suggestions of the Serovo–Glazkovo culture resulting from admixture between the preceding local Kitoi culture and an incoming ANE-rich population (de Barros Damgaard, Martiniano, et al. 2018; Yu et al. 2020), our study presents findings that challenge this hypothesis. Specifically, our analysis of the earliest Serovo–Glazkovo individual, irk030, belongs to the APS ancestry and has no direct connection with the Kitoi culture–related populations. This raises an alternative scenario that Serovo–Glazkovo culture originated from a local APS substratum possibly tracing back to the pre-Kitoi period, not from the Early Neolithic Kitoi culture, although more ancient genomes are required to verify the APS genetic profile of the earliest Serovo–Glazkovo population. Previous studies also suggested a discontinuity between the Kitoi and Serovo–Glazkovo cultures based on a gap of archeological records between them or a significantly different mitochondrial haplogroup composition (Weber et al. 2002; Mooder et al. 2006). Instead, our research indicates that region from West Baikal to Altai Mountains could be the refugium of APS ancestry, providing a promising avenue for hypothesis testing. Accurate assessment can be achieved when early Serovo–Glazkovo individuals are unearthed in this region in the future.

It is noteworthy that the Siberian ancestry found across Eurasia and North America can be traced to a single gene pool best represented by Yakutia_MN. The genetic difference between the Middle Neolithic Belkachi culture (Yakutia_MN) and Late Neolithic Ymyyakhtakh culture (Yakutia_LN) allows us to reason that the spread of the Siberian ancestry happened prior to Yakutia_LN. This fits with the time range given by the admixture date for the earliest presence of the Siberian ancestry in northeastern Europe (Bolshoy Oleni Ostrov; ca. 4,000 BP) and by the initial appearance of the Paleo-Eskimo culture (ca. 4,500 BP). This genetic evidence also aligns well with the dissemination of ceramic and lithic technologies, as documented in previous studies (Coutouly 2016; Kozlov et al. 2020). Interestingly, evidence of the second wave of Siberian ancestry expansion, associated with Ymyyakhtakh culture, is discernible in Nganasan individuals but not in another Samoyedic-speaking population, Selkup. This suggests that the divergence within Samoyedic-speaking populations may go back to the Neolithic period.

Despite our effort to construct a detailed historical model for the relationship between ancient and present-day Siberian populations, there are certain aspects that require further investigation. First, our understanding on the distribution and impact of the APS ancestry in Siberia is based only on a handful of ancient genomes, thus leaving it unknown how it became superseded by later migrant populations across Siberia. Second, Yakutia_MN and Russia_Bolshoy belong to different Y haplogroups, N and Q, respectively, which may be attributed to the limited availability of Yakutia_MN genomes. Third, we hypothesize an unsampled population whose genetic profile is similar to Yakutia_MN but has a higher genetic affinity with later Yakutia_LN, leaving it to be tested in future studies. We call for future paleogenomic studies to produce Middle Neolithic ancient genomes across Siberia, especially from Yakutia, to enhance our understanding on the details of the spread of the Siberian ancestry.

## Materials and Methods

### Genotype Data Preparation

We compiled previously published genome-wide genotype data of ancient individuals for the “1240K” panel, a set of 1,233,013 ancestry-informative single nucleotide polymorphisms (SNPs; Mathieson et al. 2015; Fu et al. 2016). First, we took publicly available random pseudo-haploid pulldown genotype data for the 1240K panel from the Allen Ancient DNA Resource v37.2 and other individual studies (supplementary table S2, Supplementary Material online; Rasmussen et al. 2010, 2014, 2015; Fu et al. 2014, 2016; Lazaridis et al. 2014, 2016, 2017; Raghavan et al. 2014, 2015; Allentoft et al. 2015; Jones et al. 2015; Mathieson et al. 2015; Jeong et al. 2016, 2018, 2020; Kılınç et al. 2016; Saag et al. 2017; Unterländer et al. 2017; Yang et al. 2017; Harney et al. 2018; Lipson et al. 2018; Mathieson et al. 2018; Mittnik et al. 2018; Moreno-Mayar et al. 2018; Narasimhan et al. 2019; Ning et al. 2020; Skourtanioti et al. 2020; Yang et al. 2020; Yu et al. 2020; Wang et al. 2021). Second, for ancient individuals whose genotype data are not available but aligned reads (BAMs) are, we obtained BAM files from the European Nucleotide Archive (https://www.ebi.ac.uk/ena), using the accession numbers provided in the original publications (de Barros Damgaard, Marchi, et al. 2018; de Barros Damgaard, Martiniano, et al. 2018; Krzewińska et al. 2018; Lamnidis et al. 2018; Flegontov et al. 2019; Sikora et al. 2019; Kılınç et al. 2021; Wang et al. 2023). Finally, we obtained FASTQ files of present-day Khanty and Nenets from the National Center for Biotechnology Information (NCBI) Sequence Read Archive (SRA; Wong et al. 2017). Supplementary tables S2 and S3, Supplementary Material online, provide a summary of the reference and raw data type (i.e. 1240K pulldown haploid genotype, BAM, or FASTQ) for each ancient genome data.

For present-day Khanty and Nenets, we aligned raw reads to the human reference genome with decoy sequence (hs37d5) using the bwa-aln and bwa-samse modules in Burrows-Wheeler Aligner (BWA; Li and Durbin 2009). Before genotyping with BAM file, we eliminated polymerase chain reaction duplicates using Picard MarkDuplicates module v2.20.0 for present-day data (https://broadinstitute.github.io/picard/) and dedup v0.12.8 for ancient data (Peltzer et al. 2016) and removed low-quality reads with Phred-scaled mapping quality score lower than 30 using SAMtools v1.9 (Li et al. 2009). We then examined the pattern of post-mortem chemical damages for ancient samples using mapDamage program v2.2.1 (Jónsson et al. 2013) to ensure that it matched the expected pattern from the reported library preparation method. To minimize the impact of chemical damages in genotyping, we trimmed 3 and 10 bps at both ends of reads for partial-UDG and non-UDG treated double-strand libraries, respectively, using bamUtils v1.0.15 (Jun et al. 2015).

Using these BAM files, we recalibrated the BAM files of Khanty and Nenets using BaseRecalibrator module and calculated genotype likelihoods for variants in the 1240K panel using UnifiedGenotyper module in GATK v3.8.1.0 (DePristo et al. 2011). Then, we calculated the genotype posterior probability with genotype likelihood and a prior of (0.9985, 0.0010, 0.0005) and took the highest probability genotype call when its probability exceeds 0.9. For ancient samples, we produced random pseudo-haploid genotype data for the 1240K panel by randomly choosing one high-quality base (Phred-scaled base quality score 30 or higher) using the pileupCaller v1.5.2 with “randomHaploid” option (https://github.com/stschiff/sequenceTools; v1.5.2 last accessed at April 19, 2023). For double-stranded library data, we used ends-masked BAM files for transition SNPs and nonmasked BAM files for transversions. Excluding bases that are enriched in post-mortem damages, end-masking substantially reduces incorporation of post-mortem damages into the genotype calls while still being unable to fully eliminate damages in double-stranded non-UDG libraries. For single-stranded library data, we used nonmasked BAM files and applied the “singleStrandMode” option: this minimizes the impact of chemical damages by disregarding forward strand reads for C/T SNPs and reverse strand reads for G/A SNPs. We intersected the 1240K genotype data of ancient individuals with two sets of present-day worldwide individuals: (i) 1240K genotype data of individuals from the Simons Genome Diversity Project (Mallick et al. 2016) and (ii) a broader set of individuals genotyped on the Affymetrix Axiome Genome-wide Human Origins 1 (“HumanOrigins”; 593,124 autosomal SNPs; Patterson et al. 2012; Lazaridis et al. 2016; Flegontov et al. 2019; Jeong et al. 2019). For allele frequency–based analyses, our primary data set of choice was the 1240K set. However, we utilized the HumanOrigins data set for PCA and allele frequency–based analyses in cases where present-day Nganasan, Enets, Selkup, Ket, and Mansi populations were included.

We extracted metainformation about key ancient genome data including latitude, longitude, sex, radiocarbon date, mean coverage, haplogroup, and relatedness from each publication. Although almost samples showed reliable results with downloaded data, we found some samples showed inappropriate mean coverage and Y haplogroup (i.e. Yakutia_MN shows relatively higher mean coverage than reported one). Thus, we manually calculated the mean coverage using qualimap v2.2.1 (Okonechnikov et al. 2016), genotyped on Y chromosome SNPs from the ISOGG using pileupCaller v1.4.0.5 option “majorityCall,” and assigned the Y haplogroup using yHaplo program (Poznik, 2016; https://github.com/alexhbnr/yhaplo; version 2016.01.08, last accessed at April 28, 2022), respectively. In addition, we calculated pairwise genotype mismatch rate between individuals who allocated into same groups to check relatedness (Kennett et al. 2017). For each first-degree pair or duplicate, we removed one of the individuals with lower sequencing coverage for further analysis.

### PCA

We conducted PCA with present-day individuals genotyped on the autosomal part of the HumanOrigins array (*n* = 593,124) using *smartpca* v18140 from EIGENSOFT v8.0.0 (Patterson et al. 2006). We used two population sets, the first including present-day Eurasian and American (2,270) and the second including Eurasian only (2,077). We projected ancient individuals not included in the PC calculation using the “lsqrproject: YES” option. Samples used in PCA are listed in supplementary table S2, Supplementary Material online.

### *f* Statistics

We calculated the *f* statistics by *qp3pop* and *f*_4_ functions from the R library ADMIXTOOLS2 v2.0.0. (https://github.com/uqrmaie1/admixtools, publication pending). We calculated outgroup-*f*_3_ using the Central African population Mbuti as an outgroup to measure shared genetic drift between target populations. Likewise, Mbuti was used as an outgroup to calculate *f*_4_ statistics in the form of *f*_4_(Mbuti, X; target1, target2) for testing symmetricity between targets or searching additional admixture sources. Populations used in *f* statistics are listed in supplementary table S2, Supplementary Material online, and the results of *f* statistics are summarized in supplementary tables S7 to S9, Supplementary Material online.

### *qpWave* and *qpAdm* Analysis

We used *qpWave* and *qpAdm* functions from the R library ADMIXTOOLS2 v2.0.0. for admixture modeling analysis. We used the following populations as a base outgroup set for both *qpWave* and *qpAdm* analyses: present-day Central African hunter–gatherers Mbuti (1240K: *n* = 5; HumanOrigins: *n* = 10), Taiwanese Aborigines Ami (1240K: *n* = 2; HumanOrigins: *n* = 10), Native Americans Mixe (1240K: *n* = 5; HumanOrigins: *n* = 10), indigenous Andamanese islander Onge (1240K: *n* = 2; HumanOrigins: *n* = 11), early Neolithic Iranians from the Ganj Dareh site Iran_N (*n* = 8; Lazaridis et al. 2016; Narasimhan et al. 2019), Epipaleolithic European Villabruna (*n* = 1; Fu et al. 2016), early Neolithic farmers from western Anatolia Anatolia_N (*n* = 23; Mathieson et al. 2015), early Neolithic northern East Asian Yumin from Inner Mongolia (*n* = 1; Yang et al. 2020), and Neolithic southern Russia West_Siberia_N (*n* = 3; Narasimhan et al. 2019). In addition, when multiple admixture models were feasible, *qpAdm* rotating approach, which systematically shifts candidates from source to outgroup, was used to find the best proximal source.

### Graph-Based Analysis

In order to test component-wise admixture models, graph-based analysis was implemented using the *qpGraph* function from the R library ADMIXTOOLS2 v2.0.0. Before graph fitting, *f*_2_ statistics between all pairs of targets were calculated by the *extract_f2* function in ADMIXTOOLS2 with the “max_miss=0” option, the same as the “allsnps: NO” option from the previous version. The number of SNPs remaining by applying this option was 182,628. Mbuti population was also used as an outgroup in this analysis, and the following populations were used for distal representatives: MA1 for ANE; WHG for Mesolithic hunter–gatherers from Europe; USR1 for Native Americans; and EastBaikal_N for ANA. Then, Middle Holocene populations were systematically added by following orders: irk030, Dzhylinda-1, WestBaikal_EN, Yakutia_MN, Saqqaq, WestBaikal_LNBA, and Yakutia_LN. The estimated branch length and admixture proportions were converted to dot file by in-house code, and we plotted admixture graph using Graphviz 6.0.1.

## Supplementary Material

evae063_Supplementary_Data

## Data Availability

Allen Ancient DNA Resource (AADR) v37.2 data sets were derived from sources in the public domain: https://reich.hms.harvard.edu/allen-ancient-dna-resource-aadr-downloadable-genotypes-present-day-and-ancient-dna-data. The public domain and accession numbers of other present-day and ancient genome data are listed in supplementary table S3, Supplementary Material online. The genotype data of ancient individuals for the 1240K panel, excluding those for whom we took publicly available 1240K panel genotype calls, have been deposited in the Edmond Data Repository of the Max Planck Society (https://edmond.mpg.de/dataset.xhtml?persistentId=doi:10.17617/3.QZBM1X). All analyses performed in this study are based on publicly available programs. Program names, versions, and nondefault options are described in the Materials and Methods section. All scripts used for the analyses presented in this study are publicly available via Github repository (https://github.com/CWJeongLab/Siberia).
