# Supplementary material for: Reconstructing the Genetic Relationship between Ancient and Present-Day Siberian Populations
Source: Genome Biol Evol. 2024 Mar 25;16(4):evae063. doi: 10.1093/gbe/evae063 (PMC10999361; doi:10.1093/gbe/evae063)
Supplement: evae063_Supplementary_Data [file evae063_supplementary_data.zip › Siberia Supplementary_Figure r1_v6.docx]

**Supplementary Information**

**Reconstructing the genetic relationship
between ancient and present-day Siberian populations**

Haechan Gill, Juhyeon Lee, Choongwon Jeong*

* Correspondence to: cwjeong@snu.ac.kr (C.J.)

**This file includes:**

Supplementary Figures S1 to S4


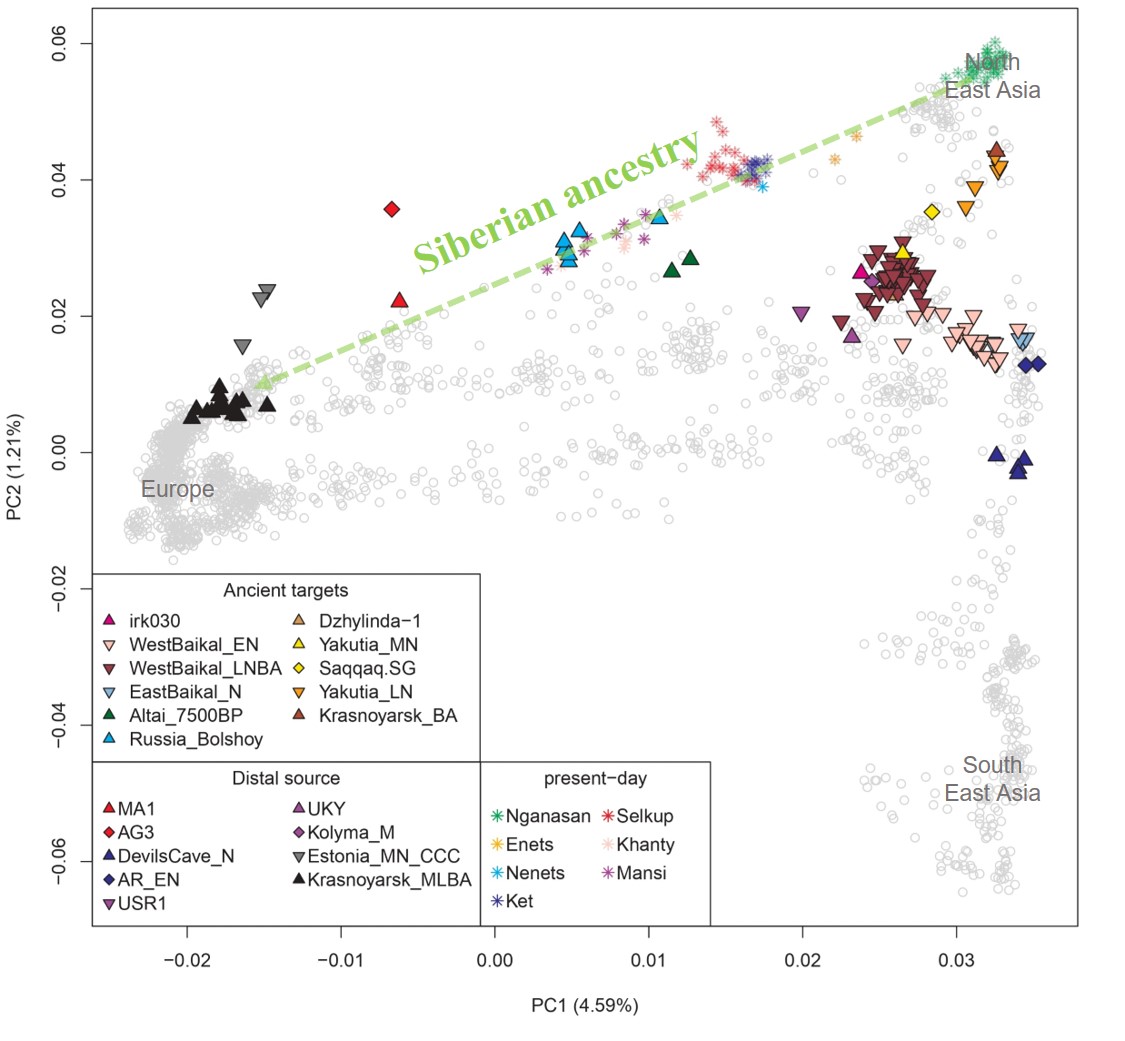


**Figure S1. Principal component analysis performed with present-day Eurasian individuals.** ​​The principal component analysis is performed with present-day Eurasian individuals, and each present-day sample is placed on principal component 1 and 2 coordinates by colored asterisk symbols (key Siberian populations) or grey circles (others). Key ancient individuals are projected on pre-calculated principal components and marked with color-filled polygon symbols.


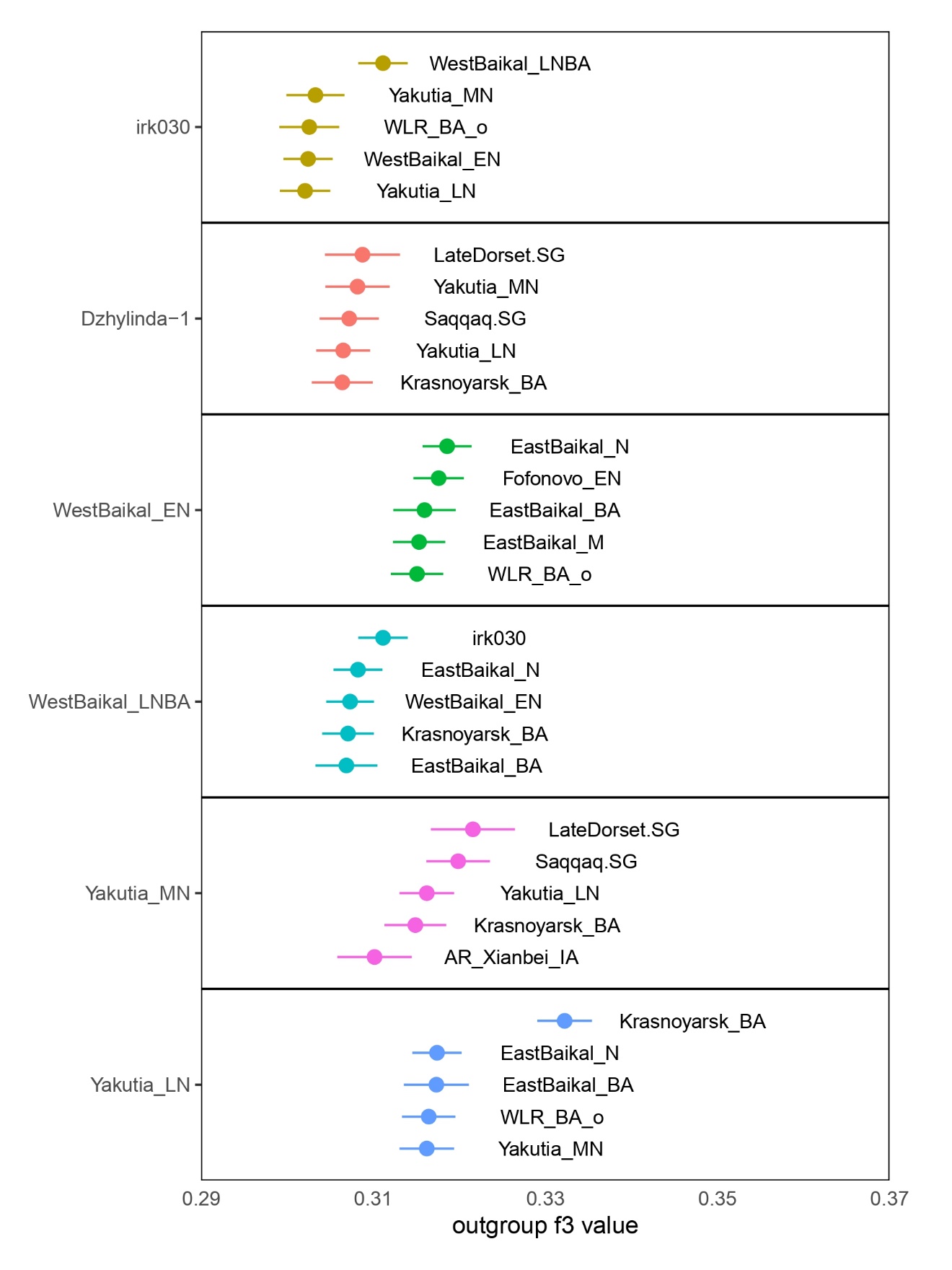


**Figure S2. Outgroup-*fз* values for the Middle Holocene Siberian populations.** Outgroup-*f*₃ statistics are calculated in the form of *f*₃(Mbuti; X, Target), and the highest 5 populations for each target are shown. Horizontal bars represent the point estimate ±1 standard error, and standard errors are calculated by 5cM block jackknifing. All outgroup-*fз* values are reported in Table S8.

**
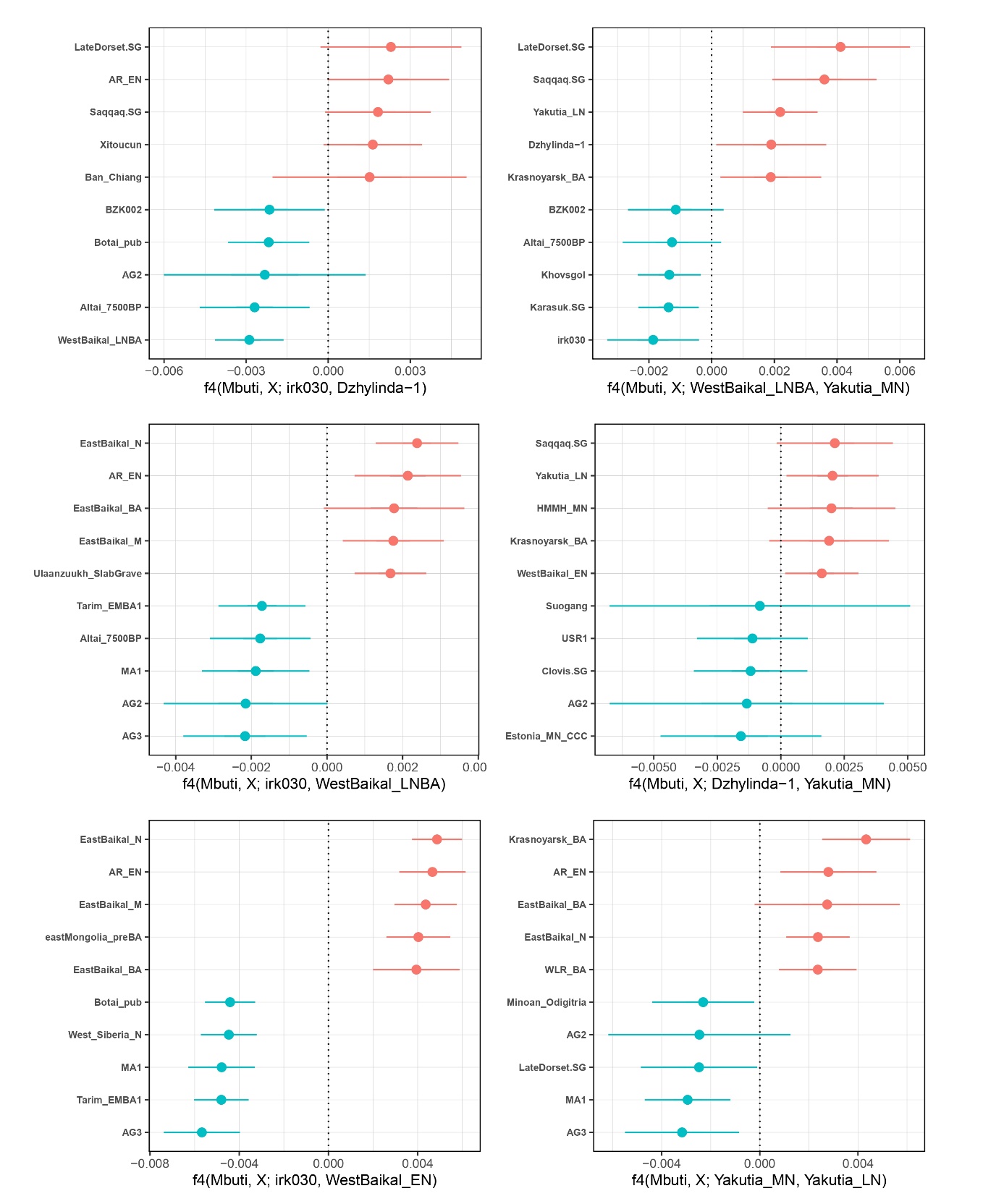
Figure S3. Genetic symmetricity test between Middle Holocene Siberians.** *F*₄ statistics in the form *f*₄(Mbuti, X; target1, target2) are calculated. The 5 most positive and 5 most negative *f*₄ statistics are shown in red and blue, respectively. Horizontal bars represent the point estimate ±3 standard error, and standard errors are calculated by 5cM block jackknifing. All *f*₄ values are reported in Table S7.


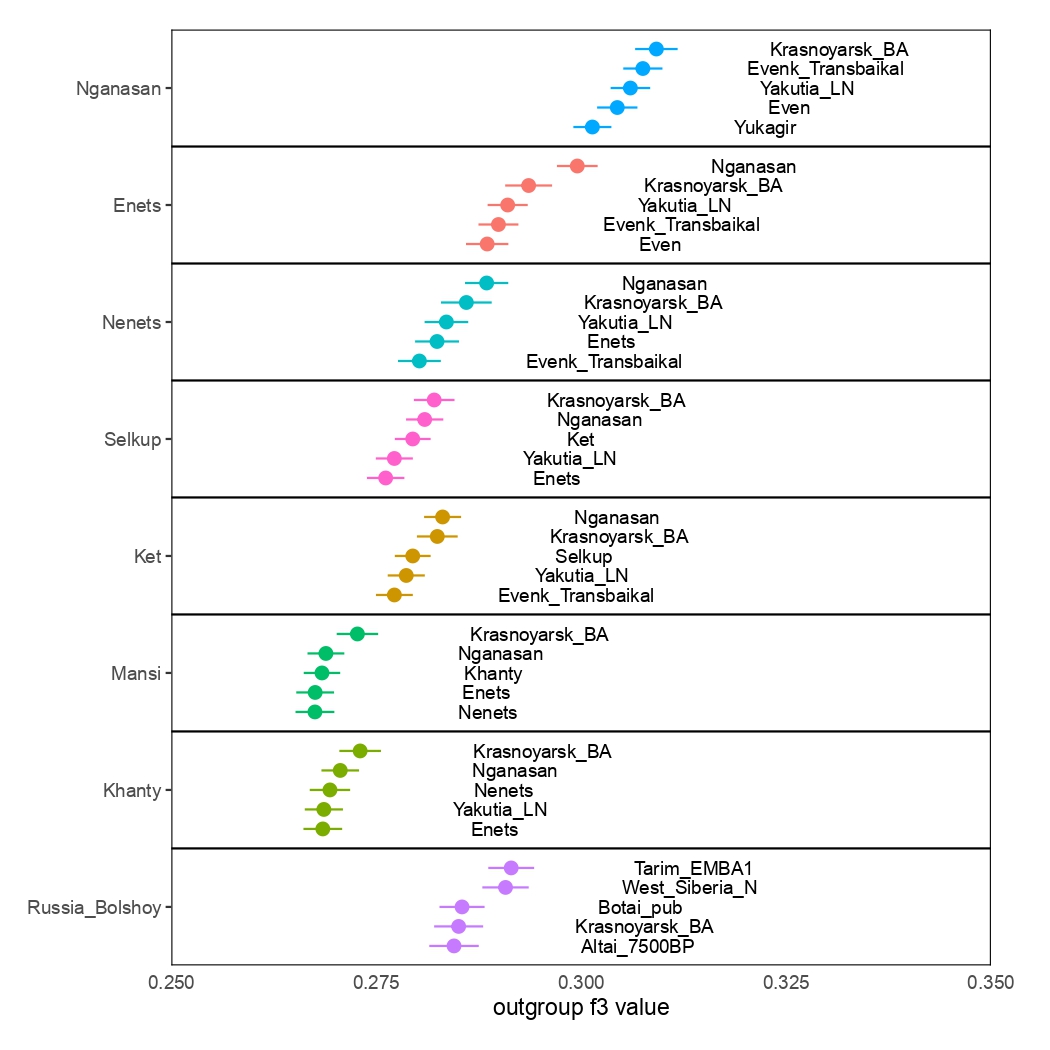


**Figure S4. Outgroup-*fз* values for the Siberian ancestry-related populations.** Outgroup-*f*₃ statistics are calculated in the form of *f*₃(Mbuti; X, Target), and the highest 5 populations for each target are shown. Horizontal bars represent the point estimate ±1 standard error, and standard errors are calculated by 5cM block jackknifing. All outgroup-*fз* values are reported in Table S8-S9.
